# The Effects of Ferulic Acid on the Growth Performance, Immune Function, Antioxidant Capacity, and Intestinal Microbiota of Broiler Chickens

**DOI:** 10.3390/genes16050572

**Published:** 2025-05-13

**Authors:** Xianguo Yi, Quanchao Ma, Zhili Li, Yuli Hu, Haigang Wu, Rui Wang, Xuyang Sun, Enen Wang, Chaofeng Ma, Qingmin Qin

**Affiliations:** 1 College of Animal Science and Technology, Xinyang Agriculture and Forestry University, Xinyang 464000, Chinasunxuyang@163.com (X.S.);; 2 Chongqing Animal Disease Prevention and Control Center, Chongqing 400120, China; huyuli2025@sina.com; 3 Xinyang Prevention and Control Center of Animal Disease, Xinyang 464000, China; machaofeng5@163.com

**Keywords:** ferulic acid, anti-inflammatory, metabolic function, broiler chickens

## Abstract

Objectives: Ferulic acid is a natural and safe herbal feed additive. This study aims to evaluate the effects of ferulic acid on the growth performance, anti-inflammatory and antioxidant capacities, immune function, and intestinal microbiota of broiler chickens. Methods: A total of 320 broiler chickens, aged 14 days, were randomly divided into four groups: a blank control group (MA group), a low-concentration ferulic acid group (BM group, 10 mg/kg), a medium-concentration ferulic acid group (CM group, 30 mg/kg), and a high-concentration ferulic acid group (DM group, 90 mg/kg) after a 14-day acclimatization period. The experiment lasted for 28 days, and the chickens were dissected on day 29. Results: The results showed that compared to the MA group, the feed-to-meat ratio in the CM and DM groups was significantly reduced. The activity of duodenal trypsin in the CM and DM groups was significantly enhanced, and the activity of pancreatic protease in the DM group was significantly increased. The serum levels of urea nitrogen, creatinine, and triglycerides were significantly elevated in the CM and DM groups. The serum malondialdehyde (MDA) levels in the BM, CM, and DM groups were significantly reduced, while the activities of superoxide dismutase (SOD) and glutathione peroxidase (GSH-Px) were significantly increased in the CM and DM groups. The serum interleukin-2 (IL-2) levels in the BM group were significantly decreased, while interferon-gamma (IFN-γ) levels in the CM group and complement component 3 (C-3) levels in the DM group were significantly increased. The mRNA expression levels of TLR4, MyD88, NF-κB, TNF-α, NLRP3, IL-1β, and IL-18 in the jejunum of the DM group were significantly reduced. The diversity of cecal microbiota in the ferulic acid groups changed, with a certain degree of increase in the relative abundance of *Spirulina* and *Ruminococcus*. The relative abundance of *Escherichia coli* in the DM group significantly increased, altering the metabolic function of the cecal microbiota in broiler chickens. Conclusions: The above results indicate that ferulic acid, as a novel feed additive for broiler chickens, has an impact on the growth performance, anti-inflammatory and antioxidant capacity, immune function, and intestinal microbiota of broiler chickens.

## 1. Introduction

Broiler chicken farming constitutes a significant portion of poultry production in China and represents a vital sector of the country’s poultry breeding industry. Antibiotics are frequently utilized as feed additives to enhance the growth performance of broiler chickens. However, this practice not only escalates feed costs but also contributes to issues related to antibiotic misuse and microbial resistance [1]. Since 2020, the Ministry of Agriculture of China has imposed a complete ban on the inclusion of growth-promoting antibiotics in feed, with the exception of traditional Chinese medicine, thereby underscoring the pressing need for alternatives to antibiotics.

Ferulic acid (FA), a type of polyphenolic phenolic acid, is one of the active components found in traditional Chinese medicinal herbs, such as ferula, angelica, and ligusticum. It is characterized by low toxicity and rapid metabolism and mainly exists in a bound state [2]. At present, some studies have found that ferulic acid plays a pharmacologically active role in relieving a variety of diseases, such as diabetes, cancer, etc. [3,4]. A recent study also found that ferulic acid may have a protective mechanism for the hearts of diabetic mice [4]. Currently, the application of ferulic acid has mainly focused on its anti-inflammatory and antioxidant functions in mice, with relatively few reports on its effects on the intestinal microbiota, anti-inflammatory, and antioxidant properties in broiler chickens [5,6].

The intestinal flora plays a key role in the digestion of feed and the absorption of nutrients, and it also has an indispensable role in the immune function, the development of intestinal structure and morphology, and the resistance to the invasion of endotoxins [7,8]. A large number of studies have shown that the intestinal microbiota is involved in the metabolism of a variety of nutrients in the host. The phylum Firmicutes can decompose carbohydrates and produce butyrate [9,10]. *Lactobacillus* can hydrolyze proteins and produce a variety of amino acids [11]. *Bifidobacterium* can participate in the metabolism of a variety of vitamins, such as vitamin K, vitamin B12, in the host [12]. In addition, the homeostasis of intestinal flora plays an important role in maintaining host health. Lactobacillus inhibits intestinal inflammation by enhancing the immune function of intestinal mucosa and can inhibit the growth of intestinal pathogens and borne pathogens [13]. Infection with Clostridium perfringens led to a decrease in the relative abundance of *Lactobacillaceae*, *Lactobacillus*, *Blautia*, and *Ruminococcaceae* [14,15]. *E. coli* can cause heart function disorders in the body, and bacterial virulence factors such as lipopolysaccharide (LPS) are the main factors causing cardiac inflammation and myocardial cell death [16]. Based on a large number of literature reports, it was concluded that the abundance of intestinal flora is closely related to the growth performance of chickens.

This study uses broiler chickens as an experimental model, adding different concentrations of ferulic acid to the feed to explore its effects on growth performance, intestinal microbiota, and immune function in broiler chickens. The aim is to provide scientific data for the application of ferulic acid and to establish foundational data for its potential as an ideal alternative to antibiotics.

## 2. Materials and Methods

### 2.1. Experimental Animals and Grouping

Ferulic acid was supplied by Changzhou Ding Yunlong Biotechnology Co., Ltd. (Changzhou, China). The experimental subjects were 14-day-old broilers obtained from the Gushi Chicken Farm in Xinyang City, Henan Province. Following a 14-day acclimatization period, 320 broilers were randomly selected as individuals of similar weight and divided into 4 groups, with 80 individuals in each group. Each group consisted of 4 replicates, with 20 individuals in each replicate. The control group (MA group, fed a basic diet), the BM group (basic diet supplemented with ferulic acid at 10 mg/kg), the CM group (basic diet supplemented with ferulic acid at 30 mg/kg), and the DM group (basic diet supplemented with ferulic acid at 90 mg/kg). Throughout the feeding period, the broilers had free access to feed and water, and the experiment continued for 28 days. All experimental procedures in this study were conducted in accordance with the “Regulations on the Administration of Experimental Animals” approved by the State Council of the People’s Republic of China.

### 2.2. Growth Performance

Record the daily feed intake of each group of broilers. On days 1, 14, and 28 of the experiment, after fasting for 12 h, randomly select 8 broilers from each group, weigh them, and allow free access to water during the fasting period. Calculate the daily feed-to-gain ratio using the formula: feed-to-gain ratio = total feed intake ÷ (total final weight − total initial weight).

### 2.3. Sample Collection

On day 29, the broiler chickens were weighed, and non-anticoagulated venous blood was collected to obtain serum. The abdominal cavity was opened, and intestinal tissue from the jejunum was collected. After rinsing the intestinal contents with cold phosphate-buffered saline (PBS), they were frozen in liquid nitrogen and stored at −80 °C. The contents of the cecum and duodenum were also collected, quickly frozen in liquid nitrogen, and stored for later use.

### 2.4. Determination of Serum Biochemical Indexes

Place the collected venous blood in a refrigerator at 4 °C and let it sit overnight. Centrifuge at 4 °C at 3000 r/min for 15 min to collect the serum. Use an automatic biochemical analyzer to test the levels of urea nitrogen, creatinine, total cholesterol, and triglycerides in the serum.

### 2.5. Determination of Antioxidant Index and Immune Factor Levels

Using the reagent kits produced by the Nanjing Institute of Bioengineering (Nanjing, China), the activity of superoxide dismutase (SOD), glutathione peroxidase (GSH-Px), and the content of malondialdehyde (MDA) in the serum of the broiler chickens was detected. The levels of IL-2, complement C-3, and IFN-γ in the serum of broiler chickens were measured using the ELISA kits produced by Shanghai Hepeng Biotechnology Co., Ltd. (Shanghai, China).

### 2.6. Determination of Digestive Enzyme Activity in Duodenum

The reagent kit produced by the Nanjing Institute of Bioengineering (Nanjing, China) was used to detect the activity of chymotrypsin, lipase, trypsin, and amylase in the contents of the duodenum.

### 2.7. RT-qPCR

Total RNA was extracted from the jejunal tissue, and the RNA concentration was measured using a micro-volume UV spectrophotometer (Shanghai, China). According to the instructions of the reverse transcription kit, mRNA was reverse transcribed into cDNA. Real-time quantitative PCR (qPCR) was performed according to the instructions of the 2 × SYBR Green qPCR Master Mix (Low ROX) kit Provided by Wuhan Saiweier Biotechnology Co., Ltd. (Wuhan, China) with the program set to 95 °C for 30 s, 95 °C for 15 s, 60 °C for 30 s, and 72 °C for 30 s, for a total of 40 cycles. The primers were synthesized by Anshida (Tianjin) Biotechnology Co., Ltd. (Tianjin, China), and the primer design can be found in Supplementary Table S1.

### 2.8. Bioinformatics Analysis of Intestinal Flora

Samples of cecal contents were sent to Wuhan Benna Technology Co., Ltd. (Wuhan, China) for high-throughput sequencing of 16S rDNA. The 16S rRNA gene was amplified using primers 515F (5′-GTGCCAGDMGCCGCGGTAA-3′) and 806R (5′-GGACTACHVGGGTWTCTAAT-3′) targeting the bacterial V3-V4 variable region. High-throughput sequencing was performed on the Illumina HiSeq 2500 platform. The raw 16S data sequences were filtered and assembled using the QIIME V1.9.0 and FLASH software (flash29.0.0) packages. High-quality sequences were compared with the Silva reference database (https://www.arb-silva.de/, accessed on 7 June 2024) and clustered into OTUs at a 97% similarity level using the UCLUST algorithm (http://drive5.com/ssu_data_analysis_service.html, accessed on 7 June 2024). Taxonomic analysis of the OTU representative sequences was conducted using the RDP classifier Bayesian algorithm. Functional metagenomic predictions for all samples were made using PICRUST v1.1.327. The functional differences between groups were compared using the *STBMP 2.1.3 t*-test.

### 2.9. Statistical Analysis

Statistical analysis was performed using GraphPadPrism: 8 software (GraphPad Software: San Diego, CA, USA, www.graphpad.com). The grouped data were analyzed using one-way ANOVA, followed by a Tukey–Kramer multiple comparison test, and the results were expressed as “mean ± standard deviation”. *p* < 0.05 is considered statistically significant, indicating a significant difference; *p* > 0.05 indicates no significant difference.

## 3. Results

### 3.1. Effects of Ferulic Acid on Production Performance of Broilers

As shown in Table 1, compared to the MA group, the feed-to-meat ratio in the BM group and CM group was significantly increased, while the DM group decreased.

### 3.2. Effect of Ferulic Acid on Serum Biochemical Indexes

As shown in Table 2, compared to the MA group, the serum levels of urea nitrogen, creatinine, total cholesterol, and triglycerides in the CM group and DM group were significantly elevated; compared to the BM group, the serum levels of urea nitrogen, creatinine, total cholesterol, and triglycerides in the CM group and DM group were also significantly elevated.

### 3.3. Effects of Ferulic Acid on Duodenal Digestive Enzyme Activity

Compared to the MA group, the CM group and DM group showed a significant increase in chymotrypsin activity, while the DM group exhibited a significant increase in both chymotrypsin and trypsin activities. The lipase activity in the DM group significantly increased, while the amylase activity in the BM group was the highest, with a significant difference, as shown in Table 3. The above results indicate that ferulic acid promotes the digestion and absorption of feed in broiler chickens by increasing the activity of duodenal digestive enzymes.

### 3.4. Effect of Ferulic Acid on Antioxidant Index and Immune Factor Levels

As shown in Table 4, compared to the MA group, the MDA content in the BM group was significantly reduced, and the MDA content in the CM group and DM group was also significantly reduced, while the activities of SOD and GSH-Px were significantly increased. As shown in Table 5, compared to the MA group, the IL-2 content in the BM group was significantly reduced, the IFN-γ content in the CM group was significantly increased, and the C-3 content in the DM group was significantly increased.

### 3.5. Effects of Ferulic Acid on Gene Expression of Jejunum Inflammation

**Figure 1:**
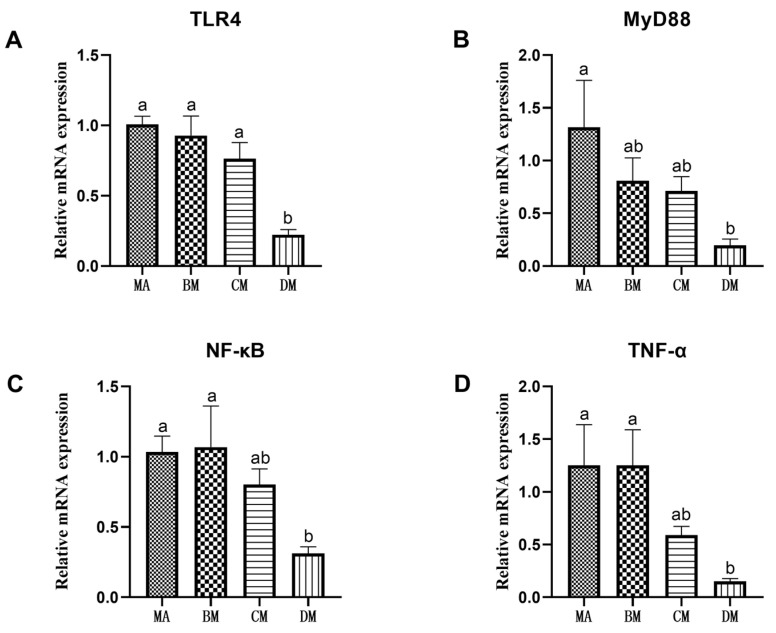
Effects of ferulic acid on the relative mRNA expression levels of (**A**) TLR4, (**B**) MyD88, (**C**) NF-κB, and (**D**) TNF-α. Note: In each histogram, bar charts with different letters indicate statistically significant differences between groups (*p* < 0.05 or *p* < 0.01); There was no significant difference in the bar charts with common letters (*p* > 0.05).

**Figure 2:**
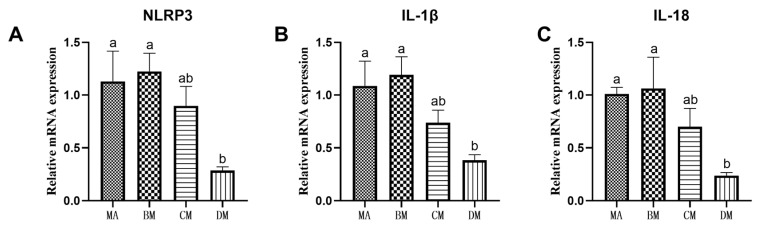
Effects of ferulic acid on the relative mRNA expression levels of (**A**) NLRP3, (**B**) IL-1β, and (**C**) IL-18. Note: In each histogram, bar charts with different letters indicate statistically significant differences between groups (*p* < 0.05 or *p* < 0.01); There was no significant difference in the bar charts with common letters (*p* > 0.05).

As shown in Figure 1 and Figure 2, compared to the MA group, the mRNA expression levels of TLR4, MyD88, NF-κB, TNF-α, NLRP3, IL-1β, and IL-18 in the DM group were significantly reduced.

### 3.6. Diversity Analysis of Intestinal Flora in Broilers

**Figure 3:**
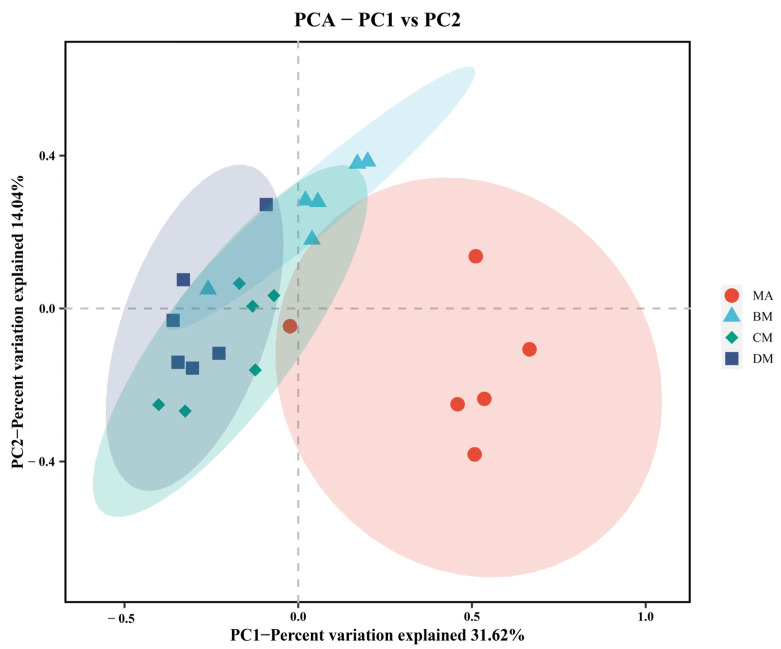
PCA analysis of cecal flora in different groups.

As shown in Table 6, compared to the MA group, there were no significant differences in the ACE index, Chao1 index, Simpson index, and Shannon index in the ferulic acid group, while the Faith_PD index in the DM group was significantly increased. As illustrated in Figure 3, there is a clear separation trend among the BM group, CM group, DM group, and MA group. The above results indicate that ferulic acid alters the diversity of the cecal microbiota.

### 3.7. Analysis of Intestinal Microflora Structure in Broilers

**Figure 4:**
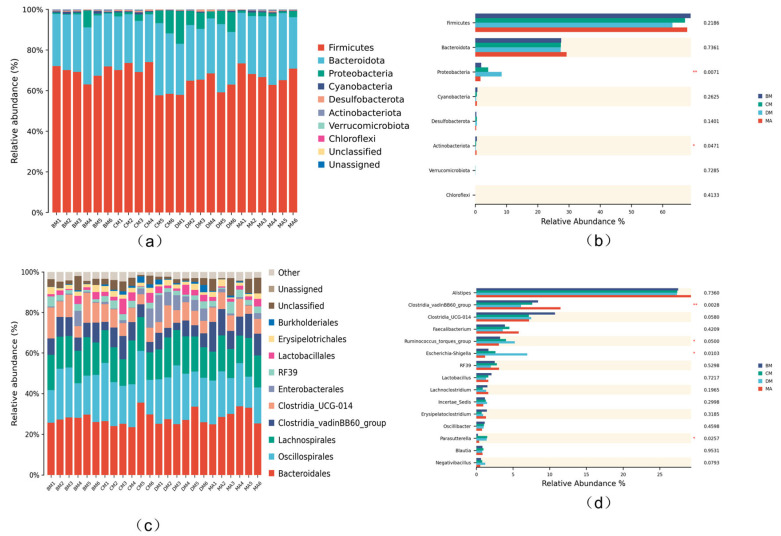
Column diagram of species composition at different levels. (**a**) Phylum level, (**b**) bar chart of species with horizontal differences in doors, (**c**) generic level, (**d**) bar chart of species with horizontal differences. * *p* < 0.05, ** *p* <0.01, respectively, compared with control group.

As shown in Figure 4a at the phylum level, *Firmicutes* and *Bacteroidota* are the dominant bacterial groups in the cecum of broiler chickens. Compared to the MA group, the relative abundance of Proteobacteria increased in the ferulic acid group. Compared with the MA group, the relative abundance of *Proteobacteria* and *Actinobacteria* in the DM group significantly increased (*p* < 0.01, *p* < 0.05, respectively) (Figure 4b). Figure 4c shows that at the genus level, the relative abundances of *Spirulina* and *Ruminococcus* increased to some extent in the ferulic acid group, while the relative abundance of *Escherichia* significantly increased in the DM group. The above results indicate that ferulic acid alters the microbial composition of the cecum. Compared with the MA group, the relative abundance of the *Clostridia_vadinBB60*_bacterial community in the experimental group was significantly reduced (*p* < 0.05), the relative abundance of *RF39* in the DM group was significantly reduced (*p* < 0.05), and the relative abundance of *Ruminococcus* and *Escherichia Shigella genera* was significantly increased (*p* < 0.05) (Figure 4d).

### 3.8. Comparison of Different Species of Intestinal Microflora in Broiler Chickens

**Figure 5:**
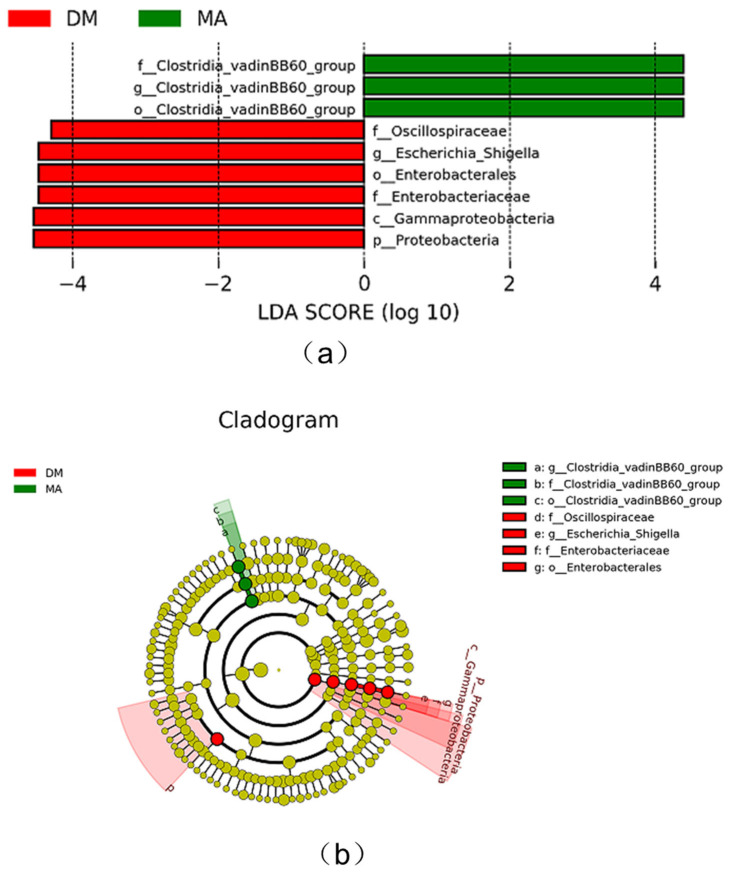
Distribution histogram based on LDA value (**a**) and evolutionary branch graph (**b**) of fecal microbiota.

As shown in Figure 5a, the LDA plot displays significant differences in species that exceed the preset values among the groups, specifically between the MA group and the DM group. The *Clostridia_vadinBB60_*group is significantly enriched in relative abundance in the blank group (MA group), while the *Oscillospiraceae*, *Escherichia*_*Shigella*, *Enterobacterales*, *Gammaproteobacteria*, and *Proteobacteria* are significantly enriched in relative abundance in the high-dose group (DM group). As shown in Figure 5b, when comparing the DM group with the MA group at the genus level, there is a significant difference in the abundance of the *Clostridia_vadinBB60_*group (*p* < 0.05).

### 3.9. Function Prediction Analysis

**Figure 6:**
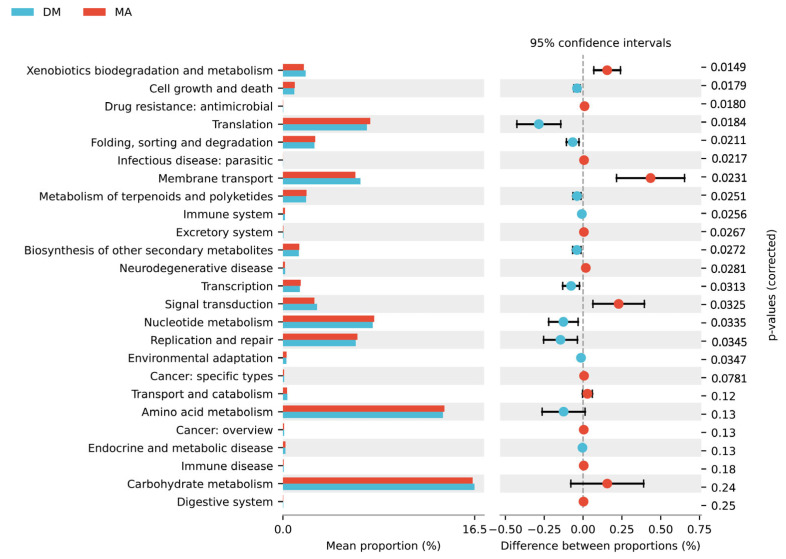
KEGG second-level functional difference histogram.

Using PICRUSt2 software (V2.5.2) to analyze the differences in the KEGG metabolic pathways, as shown in Figure 6, compared to the MA group, the functional genes of the microbial community in the DM group significantly increased in metabolic pathways such as xenobiotics biodegradation and metabolism, membrane transport, excretory system, and signal transduction. In contrast, there was a significant decrease in metabolic pathways related to cell growth and death, translation, folding, sorting and degradation, metabolism of terpenoids and polyketides, transcription, nucleotide metabolism, and replication and repair (*p* < 0.05). These results indicate that feeding high doses of ferulic acid alters the metabolic functions of the cecal microbiota in broiler chickens.

## 4. Discussion

Urea nitrogen and creatinine levels are commonly used as indicators of kidney function. Previous studies have found that intraperitoneal injection of ferulic acid in mice resulted in decreased levels of creatinine and urea nitrogen, along with increased excretion [5]. However, the results of this experiment indicate that the addition of ferulic acid to the diet significantly increased the levels of creatinine and urea nitrogen in the serum of broiler chickens, which contradicts their findings. Urea nitrogen and creatinine are major byproducts of protein metabolism in the body and can be easily influenced by dietary factors [17]. Ferulic acid may enhance protein metabolism, leading to an increase in urea nitrogen and creatinine levels. Additionally, total cholesterol and triglyceride levels in the blood are commonly used to assess lipid absorption and can reflect lipid metabolism [18]. The results of this experiment demonstrate that the total cholesterol and triglyceride levels in both the CM and DM groups were significantly elevated, indicating that ferulic acid can enhance lipid metabolism in broiler chickens.

Growth performance is the most intuitive indicator for assessing the economic benefits of broiler chicken farming; a lower feed-to-meat ratio signifies better growth performance [19]. Natural plant polyphenols have a significant positive effect on animal growth performance. Related research indicated that incorporating an appropriate amount of ferulic acid into broiler feed can significantly improve growth performance [20]. Similarly, Lin et al. found that adding a fermentation enzyme powder rich in ferulic acid derived from fungi to broiler feed also significantly decreased the feed-to-meat ratio in the initial growth phases [21]. This experiment demonstrates that the inclusion of high doses of ferulic acid in the feed can lower the feed-to-meat ratio in broilers, but the difference is not significant. The activity of intestinal digestive enzymes reflects the digestive and absorption capacity of the intestines, and the growth and development of animals are closely related to the strength of the digestive enzyme activity [22]. The results of this experiment indicate that adding ferulic acid to the feed can significantly enhance the activity of trypsin and chymotrypsin in the duodenum. This suggests that ferulic acid can boost the activity of digestive enzymes in broilers and improve intestinal digestion and absorption capacity, thereby promoting feed conversion efficiency, reducing the feed-to-meat ratio, and enhancing growth performance.

The physiological and metabolic activities of poultry are significantly influenced by heat stress, which can lead to oxidative stress and inflammatory responses [23]. Therefore, it is crucial to enhance the antioxidant capacity and immune function of broilers in farming practices. Superoxide dismutase (SOD) and glutathione peroxidase (GSH-Px) are integral components of the body’s antioxidant system, capable of improving the oxidative stress resistance of broilers and mitigating the damage caused by oxidative stress [24]. Malondialdehyde (MDA) serves as an indicator of lipid peroxidation in the body and can indirectly reflect the extent of cellular damage [25]. Wei et al. demonstrated that ferulic acid can prevent the increase in MDA levels and alleviate the decline in SOD activity [26]. Liu et al. found that administering ferulic acid reduced MDA content in the serum of Jilin white geese subjected to lipopolysaccharide-induced oxidative stress [27]. Additionally, Li et al. discovered that incorporating ferulic acid into the silage feed of goats enhanced the activity of serum antioxidant enzymes, such as total antioxidant capacity (T-AOC), SOD, and GSH-Px, in dairy goats [28]. The results of this experiment indicate that ferulic acid can enhance the activity of SOD and GSH-Px while reducing the MDA content in serum, which aligns with previous findings.

IL-2, a growth factor for immune cells in the body, exerts a pleiotropic effect [29]. During acute infections, elevated levels of IL-2 promote the development of effector T cells; however, during chronic infections and cancer, high levels of IL-2 may lead to T cell apoptosis. IFN-γ is a hallmark cytokine produced by Th1-type CD4+ cells, which play crucial roles in antitumor activity and immune regulation [30]. Complement C3 is primarily synthesized by the liver, where it is capable of lysing bacteria and viruses, and it participates in the immune response, helping to regulate the immune system [31]. The results of this experiment indicate that following the addition of ferulic acid, the serum IL-2 levels in broiler chickens decreased, while the levels of complement C3 and IFN-γ increased compared to the control group.

TLR4, a protein that initiates the inflammatory response, can promote a cascade of inflammatory reactions and play a pivotal role in regulating a series of inflammatory events in the body [32]. Upon activation, TLR4 triggers the expression of key downstream target proteins, leading to the activation and translocation of NF-κB into the nucleus. This process, in turn, promotes the assembly and activation of NLRP3, resulting in the upregulation of inflammatory factors such as TNF-α, IL-1β, and IL-18 [33,34]. Studies have shown that ferulic acid can inhibit intestinal inflammation by downregulating the expression of related inflammatory factors in the TLR4-NF-κB-NLRP3 signaling pathway, thereby exerting anti-inflammatory effects [35]. Manas Kinra and colleagues found that within the appropriate dosage range, ferulic acid effectively blocks the activation of the NLRP3 inflammasome pathway, reduces the release of IL-1β, and decreases the likelihood of disease occurrence [36]. Zhang et al. demonstrated that ferulic acid can inhibit apoptosis by lowering the levels of IL-1β, TLR4, MyD88, p-NF-κB, and p-p38MAPK, thereby reducing neuronal cell apoptosis and inflammatory infiltration [37]. Relative studies found that in vivo administration of ferulic acid alleviated intestinal damage in ulcerative colitis (UC) rats and suppressed the expression of inflammatory factors TNF-α, IL-12, and IL-1β, indicating that ferulic acid possesses significant anti-inflammatory activity [38]. The results of this experiment show that adding 90 mg/kg of ferulic acid to the diet can significantly down regulate the expression of the TLR4, MyD88, NF-κB, and NLRP3 genes, as well as the expression of TNF-α, IL-1β, and IL-18.

The gut microbiota plays a crucial role in the healthy growth and development of livestock and poultry, as it facilitates metabolic decomposition, regulates immune function, and helps resist pathogenic microorganisms. In this experiment, significant differences were observed in the alpha and beta diversity of the cecal contents of broilers. In the ferulic acid group, the abundance of *cyanobacteria* (*Spirulina*) and the genus *Ruminococcus* from the Firmicutes phylum increased to a certain extent, leading to an elevated ratio of *Firmicutes* to *Bacteroidetes*. Li et al. found that Spirulina polysaccharides can alter the relative abundance of Firmicutes, Bacteroidetes, Proteobacteria, and Actinobacteria at the phylum level, significantly improving the gut microecology of mice. This indicates that ferulic acid can promote the growth and colonization of beneficial gut bacteria to some extent [2]. Previous studies have discovered that when probiotics dominate the gut microbiota, the microorganisms metabolize to produce substances such as propionate and butyrate, which can enhance the activity of immune cells [39]. Zhang et al. demonstrated a correlation between changes in the ratio of Firmicutes to Bacteroidetes and health indices [40]. Additionally, Deng et al. used ferulic acid to intervene in a lipopolysaccharide-induced anxiety model in mice and found a significant increase in the ratio of *Firmicutes* to *Bacteroidetesc* [41].

The heatmap results indicate that, compared to the control group, the group treated with ferulic acid exhibited a significant increase in the genera *Ruminococcus* from the *Firmicutes* phylum and *Escherichia* from the *Proteobacteria phylum*. Research suggests that *Ruminococcus* plays a crucial role in the digestion of resistant starch and is also linked to intestinal diseases, autoimmune disorders, and neurological conditions [42,43]. LEfSe analysis further confirmed that following the addition of ferulic acid to the feed, there was a reduction in the subgenus *Ruminococcus_vadinBB60* within the *Firmicutes* community. A study by Dominianni et al. found that the relative abundance of *Ruminococcus* is associated with inflammatory bowel disease [44]. *Ruminococcus* is regarded as a potential pathogen and a contributor to economic losses in livestock and poultry.

Through the PICRUSt system for gene annotation of the intestinal microbiota in broilers, we found that ferulic acid significantly increased the relative abundance of the “amino acid metabolism” and “carbohydrate metabolism” pathways in KEGG functional predictions.

## 5. Conclusions

In summary, incorporating ferulic acid into the standard diet of broiler chickens can enhance their production performance and anti-inflammatory and antioxidant properties, improve immune function, and optimize the microbial composition of the cecum. This provides a theoretical foundation for the application of ferulic acid in poultry nutrition.

## Figures and Tables

**Table 1:** Effects of different concentrations of ferulic acid on production performance of broilers.

Group	MA	BM	CM	DM
average daily feed intake	40.36 ± 0.48 ^a^	39.47 ± 0.44 ^a^	39.47 ± 0.44 ^a^	32.84 ± 0.51 ^a^
average daily gain	35.92 ± 0.52 ^a^	32.03 ± 0.30 ^a^	29.71 ± 0.20 ^a^	31.67 ± 0.41 ^a^
feed–gain ratio	1.12 ± 0.07 ^b^	1.27 ± 0.14 ^a^	1.32 ± 0.10 ^a^	1.05 ± 0.03 ^b^

Note: Different lowercase letters following the data indicate significant differences (*p* < 0.05), and no letters or the same lowercase letters indicate no significance (*p* > 0.05).

**Table 2:** Serum biochemical indexes of broilers.

Group	Urea Nitrogen	Creatinine	Total Cholesterol	Triglyceride
MA	0.76 ± 0.07 ^b^	1.50 ± 1.00 ^b^	3.43 ± 0.39 ^b^	0.47 ± 0.14 ^b^
BM	0.82 ± 0.09 ^b^	1.75 ± 1.25 ^b^	4.62 ± 0.77 ^b^	0.53 ± 0.13 ^b^
CM	1.66 ± 0.01 ^a^	133.00 ± 27.38 ^a^	7.46 ± 0.26 ^a^	2.7 ± 0.12 ^a^
DM	1.74 ± 0.51 ^a^	144.25 ± 21.53 ^a^	7.54 ± 0.29 ^a^	2.82 ± 0.22 ^a^

Note: Different lowercase letters following the data indicate significant differences (*p* < 0.05), and no letters or the same lowercase letters indicate no significance (*p* > 0.05).

**Table 3:** Digestive enzyme activity of broilers.

Group	Chymotrypsin	Lipase	Trypsin	Amylase
MA	0.23 ± 0.15 ^b^	5.16 ± 1.07 ^b^	167.52 ± 40.57 ^b^	3343.33 ± 465.93 ^b^
BM	0.31 ± 0.32 ^b^	5.14 ± 1.10 ^b^	460.71 ± 27.03 ^b^	4051.33 ± 302.93 ^a^
CM	2.11 ± 0.01 ^a^	6.04 ± 2.46 ^b^	544.39 ± 39.37 ^b^	2940.00 ± 238.16 ^b^
DM	3.09 ± 0.24 ^a^	9.55 ± 1.42 ^a^	1241.91 ± 17.76 ^a^	3045.00 ± 318.56 ^b^

Note: Different lowercase letters following the data indicate significant differences (*p* < 0.05), and no letters or the same lowercase letters indicate no significance (*p* > 0.05).

**Table 4:** Effects of different concentrations of ferulic acid on antioxidant indexes in serum of broilers.

Item	MA	BM	CM	DM
SOD (U/mL)	13.18 ± 0.90 ^b^	13.68 ± 1.72 ^b^	16.98 ± 1.38 ^a^	18.31 ± 1.64 ^a^
MDA (nmol/mL)	4.46 ± 0.18 ^a^	3.9 ± 0.26 ^b^	3.8 ± 0.13 ^b^	3.52 ± 0.22 ^b^
GSH-Px (U/mL)	210.07 ± 15.46 ^b^	214.82 ± 19.65 ^b^	360.79 ± 16.58 ^a^	330.50 ± 17.26 ^a^

Note: Different lowercase letters following the data indicate significant differences (*p* < 0.05), and no letters or the same lowercase letters indicate no significance (*p* > 0.05).

**Table 5:** Effects of different concentrations of ferulic acid on immune factors in serum of broilers.

Item	MA	BM	CM	DM
IL-2 (pg/mL)	389.08 ± 40.88 ^a^	343.23 ± 25.25 ^b^	356.51 ± 29.77 ^ab^	381.20 ± 36.70 ^ab^
C-3 (µg/mL)	627.13 ± 63.92 ^b^	629.70 ± 155.61 ^ab^	632.68 ± 121.64 ^ab^	787.56 ± 175.88 ^a^
IFN-γ (pg/mL)	106.84 ± 11.34 ^b^	109.07 ± 10.61 ^ab^	122.68 ± 12.63 ^a^	109.71 ± 11.60 ^ab^

Note: Different lowercase letters following the data indicate significant differences (*p* < 0.05), and no letters or the same lowercase letters indicate no significance (*p* > 0.05).

**Table 6:** Alpha diversity of cecal flora in different groups.

Group	Faith_PD Index	ACE Index	Chao1 Index	Shannon Index	Simpson Index
MA	29.98 ± 3.22 ^b^	624.03 ± 42.80	619.71 ± 41.28	6.37 ± 0.33	0.94 ± 0.03
BM	32.71 ± 2.10 ^ab^	816.82 ± 34.94	811.75 ± 32.89	7.02 ± 0.12	0.97 ± 0.01
CM	32.81 ± 2.33 ^ab^	738.11 ± 21.55	734.50 ± 26.02	6.59 ± 0.13	0.96 ± 0.01
DM	34.47 ± 1.60 ^a^	682.91 ± 12.68	680.76 ± 13.73	6.57 ± 0.37	0.96 ± 0.01

Note: Different lowercase letters following the data indicate significant differences (*p* < 0.05), and no letters or the same lowercase letters indicate no significance (*p* > 0.05).

## Data Availability

Please contact the corresponding author if you would like access to the data supporting these findings.

## Supplementary Materials

The following supporting information can be downloaded at: https://www.mdpi.com/article/10.3390/genes16050572/s1, Table S1: Primer pairs for qRT-PCR.
