# Supplementary material for: The Effects of Ferulic Acid on the Growth Performance, Immune Function, Antioxidant Capacity, and Intestinal Microbiota of Broiler Chickens
Source: Genes (Basel). 2025 May 13;16(5):572. doi: 10.3390/genes16050572 (PMC12110936; doi:10.3390/genes16050572)
Supplement: Supplementary file 1 [file genes-16-00572-s001.zip › genes-3567660-supplementary.pdf]

The Effects of Ferulic Acid on the Growth Performance, Immune Function, Antioxidant Capacity, and Intestinal Microbiota of Broiler Chickens

Xianguo Yi [1], Quanchao Ma [1], Zhili Li [1], Yuli Hu [2], Haigang Wu [1], Rui Wang [1], Xuyang Sun [1], Enen Wang [1], Chaofeng Ma [3] and Qingmin Qin [1*]

1.College of Animal Science and Technology, Xinyang Agriculture and Forestry University, Xinyang 464000, China; sunxuyang@163.com (X.S.)

2.Chongqing Animal Disease Prevention and Control Center, Chongqing 400120, China; huyuli2025@sina.com

3.Xinyang Prevention and Control Center of Animal Disease, Xinyang 464000, China; machaofeng5@163.com

Supplementary Table S1. Primer pairs for qRT-PCR

Gene	Primer sequence (5′-3′)	Accession number
β-Actin	GAGAAATTGTGCGTGACATCA	NM_205518.2
	CCTGAACCTCTCATTGCCA	
NF-κB	CAGCCCATCTATGACAACCG	NM_001396038.1
	TCAGCCCAGAAACGAACCTC	
TNF-α	GCCCTTCCTGTAACCAGATG	XM_046927265.1
	ACACGACAGCCAAGTCAACG	
IL-1β	TCTTCTACCGCCTGGACAGC	XM_046931582.1
	TAGGTGGCGATGTTGACCTG	
IL-18	AGGTGAAATCTGGCAGTGGAAT	XM_046932263.1
	TGAAGGCGCGGTGGTTT	
MyD88	TGATGCCTTCATCTGCTACTG	XM_046910878.1
	TCCCTCCGACACCTTCTTTCTA	
NLRP3	GCTCCTTGCGTGCTCTAAGACC	XM_046918112.1
	TTGTGCTTCCAGATGCCGTCAG	
TLR4	ATCTTTCAAGGTGCCACATC	NM_001030693.2
	GGATATGCTTGTTTCCACCA	
